# PKCγ-Mediated Phosphorylation of CRMP2 Regulates Dendritic Outgrowth in Cerebellar Purkinje Cells

**DOI:** 10.1007/s12035-020-02038-6

**Published:** 2020-08-29

**Authors:** Sabine C. Winkler, Etsuko Shimobayashi, Josef P. Kapfhammer

**Affiliations:** Anatomical Institute, Department of Biomedicine, University of Basel, Pestalozzistrasse 20, CH - 4056 Basel, Switzerland

**Keywords:** Purkinje cell dendritic development, Protein kinase C gamma, Spinocerebellar ataxia type 14, Collapsin response mediator protein 2

## Abstract

The signalling protein PKCγ is a major regulator of Purkinje cell development and synaptic function. We have shown previously that increased PKCγ activity impairs dendritic development of cerebellar Purkinje cells. Mutations in the protein kinase Cγ gene (PRKCG) cause spinocerebellar ataxia type 14 (SCA14). In a transgenic mouse model of SCA14 expressing the human S361G mutation, Purkinje cell dendritic development is impaired in cerebellar slice cultures similar to pharmacological activation of PKC. The mechanisms of PKCγ-driven inhibition of dendritic growth are still unclear. Using immunoprecipitation-coupled mass spectrometry analysis, we have identified collapsin response mediator protein 2 (CRMP2) as a protein interacting with constitutive active PKCγ(S361G) and confirmed the interaction with the Duolink™ proximity ligation assay. We show that in cerebellar slice cultures from PKCγ(S361G)-mice, phosphorylation of CRMP2 at the known PKC target site Thr555 is increased in Purkinje cells confirming phosphorylation of CRMP2 by PKCγ. miRNA-mediated CRMP2 knockdown decreased Purkinje cell dendritic outgrowth in dissociated cerebellar cultures as did the transfection of CRMP2 mutants with a modified Thr555 site. In contrast, dendritic development was normal after wild-type CRMP2 overexpression. In a novel knock-in mouse expressing only the phospho-defective T555A-mutant CRMP2, Purkinje cell dendritic development was reduced in dissociated cultures. This reduction could be rescued by transfecting wild-type CRMP2 but only partially by the phospho-mimetic T555D-mutant. Our findings establish CRMP2 as an important target of PKCγ phosphorylation in Purkinje cells mediating its control of dendritic development. Dynamic regulation of CRMP2 phosphorylation via PKCγ is required for its correct function.

**Electronic supplementary material:**

The online version of this article (10.1007/s12035-020-02038-6) contains supplementary material, which is available to authorized users.

## Introduction

The protein kinase C family comprises several isoforms of serine/threonine kinases expressed in a variety of tissues where they are involved in a multitude of signalling cascades. PKCγ is one of the classical PKC isozymes, which are activated by binding diacylglycerol and calcium. It is expressed exclusively in neurons of the central nervous system and in the cerebellum where it is strongly and specifically expressed in Purkinje cells [1]. We have previously shown that activation of PKC via PMA causes a reduction in dendritic outgrowth of cerebellar Purkinje cells in organotypic slice cultures [2]. In contrast, inhibition of PKC in culture or knockout of PKCγ leads to an increased dendritic tree expansion [2, 3] showing that PKCγ is a powerful mediator of dendritic development in cerebellar Purkinje cells.

Interestingly, mutations in the PRKCG gene coding for PKCγ are associated with Purkinje cells loss and impaired motor functions in spinocerebellar ataxia 14 (SCA14). Spinocerebellar ataxias (SCAs) are disorders defined by progressive motor dysfunction along with atrophy of the cerebellum. Up to now, more than 40 mutations in the PRKCG gene are known to cause SCA14 [4, 5]. Most of these mutations were shown to have an increased kinase activity in vitro [6, 7]. In a transgenic mouse model in which the human S361G point mutation associated with SCA14 is expressed specifically in cerebellar Purkinje cells (PKCγ(S361G)-mice) [8], the mice develop mild symptoms of cerebellar ataxia and show decreased dendritic development of Purkinje cells throughout the cerebellum [9], especially in lobule VII [8]. In organotypic slice cultures prepared from these mice, Purkinje cell dendritic trees develop a stunted morphology with thickened branches [8], which is similar to that seen after pharmacological stimulation of PKC [2, 10]. These findings indicate that the S361G-mutation leads to increased activity of PKCγ [11]. However, little is known about the molecular mechanisms by which PKCγ regulates dendritic growth in Purkinje cells.

In this study, we used immunoprecipitation-coupled mass spectrometry analysis for identifying potential interactors of PKCγ in the cerebellum and identified collapsin response mediator protein 2 (CRMP2) as a potential candidate. CRMP2 belongs to a family of five proteins (CRMP1–5), which share about 75% sequence homology, with the exception of CRMP5 only sharing ~ 50% homology with the others [12, 13]. CRMP family members are known to form homo- and heterotetramers, which may enact distinct functions depending on the composition [13–15]. CRMP2 is predominantly expressed in the central nervous system where it reaches its peak early after birth, while it remains expressed during adulthood [15]. In the cerebellum, it is expressed in oligodendrocytes, granule cells and Purkinje cells, where it is localized throughout the cell [16]. Functions of CRMP2 include the regulation of cell polarization, neurite outgrowth and intracellular trafficking via the association to microtubules [17–20], which is largely modulated by posttranslational modifications such as SUMOylation, O-GlcNAcylation or phosphorylation [21–24].

We found that phosphorylation of CRMP2 is increased in organotypic slice cultures from PKCγ(S361G)-mice. When CRMP2 was knocked down or a phospho-mimetic or phospho-defective CRMP2 mutant was transfected into Purkinje cells, dendritic development of Purkinje cells in dissociated cerebellar cultures was impaired. The impairment of dendritic growth was also seen in Purkinje cells from a novel CRMP2 knock-in mouse with a phospho-defective T555A mutation of CRMP2 and could be rescued by transfection of normal CRMP2. Our results suggest that CRMP2 is an important modulator of dendritic development in cerebellar Purkinje cells and that its function depends critically upon the regulation of phosphorylation at Thr555.

## Materials and Methods

### Immunoprecipitation

The whole cerebellum from PKCγ-S361G transgenic mice was homogenized in ice-cold RIPA buffer with added protease- and phosphatase-inhibitors (Roche, Mannheim, Germany) (50 mM Tris-HCl, pH 7.4; 0.15 M NaCl, 0.25% deoxycholic acid/sodium deoxycholate, 1% NP-40, 1 mM EDTA). Samples were homogenized using an ultrasound probe and then centrifuged at 14500*g* for 15 min. Immunoprecipitations were performed using the Pierce® Crosslink Immunoprecipitation Kit (Thermo Fisher Scientific, Rockford, USA) according to standard protocols. Briefly, 5 μg of primary rabbit anti-CRMP2 (Sigma-Aldrich, St. Louis, USA; C2993), rabbit anti-PKCγ (Santa Cruz Biotechnology, Santa Cruz, USA; sc-211) or normal rabbit IgG (Santa Cruz, Santa Cruz, USA; sc-2027) was incubated with protein A/G-agarose beads for 1 h at room temperature. The antibodies were crosslinked to the beads for 30 min using disuccinimidyl suberate. Lysates were precleared with control agarose resin for 1 h at 4 °C. Seven hundred fifty micrograms of protein in 400 μL immunoprecipitation buffer was incubated with each type of antibody-bead overnight at 4 °C on a rotating platform. Beads were washed 6x with IP buffer and proteins were eluted under acidic conditions. The pH was neutralized with 1 M Tris buffer. Samples were mixed in 2x Laemmli buffer (Sigma-Aldrich, Buchs, Switzerland) and processed via SDS-PAGE and Western blotting.

### LC-MS/MS Identification and Total Protein Quantification

Immunoprecipitates were prepared as described and used for shotgun LC-MS experiments. Eluted proteins were reduced with 5 mM TCEP for 10 min at 95 °C, alkylated with 10 μM chloroacetamide for 30 min in the dark at room temperature. Sample were diluted with 0.1 M ammoniumbicarbonate solution to a final concentration of 1% sodium deoxycholate before digestion with trypsin (Promega) at 37 °C overnight (protein to trypsin ratio: 50:1). After digestion, the samples were supplemented with TFA to a final concentration of 0.5% and HCl to a final concentration of 50 mM. Precipitated sodium deoxycholate was removed by centrifugation (15 min at 4 °C at 14,000 rpm). Then, peptides were desalted on C18 reversed phase spin columns according to the manufacturer’s instructions (Macrospin, Harvard Apparatus), dried under vacuum and stored at − 80 °C until further processing. One microgram of peptides of each sample was subjected to LC–MS analysis using a dual pressure LTQ-Orbitrap Elite mass spectrometer connected to an electrospray ion source (both Thermo Fisher Scientific) as described recently [25] with a few modifications. In brief, peptide separation was carried out using an EASY nLC-1000 system (Thermo Fisher Scientific) equipped with a RP-HPLC column (75 μm × 30 cm) packed in-house with C18 resin (ReproSil-Pur C18–AQ, 1.9 μm resin; Dr. Maisch GmbH, Ammerbuch-Entringen, Germany) using a linear gradient from 95% solvent A (0.15% formic acid, 2% acetonitrile) and 5% solvent B (98% acetonitrile, 0.15% formic acid) to 28% solvent B over 90 min at a flow rate of 0.2 μl/min. The data acquisition mode was set to obtain one high resolution MS scan in the FT part of the mass spectrometer at a resolution of 120,000 full width at half-maximum (at m/z 400) followed by MS/MS scans in the linear ion trap of the 20 most intense ions using rapid scan speed. The charged state screening modus was enabled to exclude unassigned and singly charged ions and the dynamic exclusion duration was set to 30 s. The ion accumulation time was set to 300 ms (MS) and 25 ms (MS/MS).

For label-free quantification, the generated raw files were imported into the Progenesis QI software (Nonlinear Dynamics (Waters), Version 2.0) and analysed using the default parameter settings. MS/MS-data were exported directly from Progenesis QI in mgf format and searched against a decoy database the forward and reverse sequences of the predicted proteome from *Mus musculus* (UniProt, download date: 16/11/2015, total of 33,984 entries) using MASCOT (version 2.4.1). The search criteria were set as follows: full tryptic specificity was required (cleavage after lysine or arginine residues); 3 missed cleavages were allowed; carbamidomethylation (C) was set as fixed modification; oxidation (M) as variable modification. The mass tolerance was set to 10 ppm for precursor ions and 0.6 Da for fragment ions. Results from the database search were imported into Progenesis QI and the final peptide measurement list containing the peak areas of all identified peptides, respectively, was exported. This list was further processed and statistically analysed using the SafeQuant R script developed at the Proteomics Core Facility at Biozentrum, University of Basel [25]. The peptide and protein false discovery rate (FDR) was set to 1% using the number of reverse hits in the dataset.

### Proximity Ligation Assay (Duolink)

The Duolink proximity ligation assay was used to determine the interaction between proteins following the manufacturer’s protocols. HeLa cells (RRID: CVCL_0030; ATCC, Molsheim Cedex, France) were transfected with PKCγ using Effectene (Qiagen, Hilden, Germany) and fixed after 24 h with 4% PFA. Primary antibodies rabbit anti-CRMP2 (1:1000; Sigma-Aldrich; C2993), mouse anti-PKCγ (PKC66; 1:500; Invitrogen; 13-3800) and mouse anti-α-tubulin (DM1A; 1:1000; Invitrogen; 14-4502-82) were used and labelled with the PLA probes anti-rabbit minus (Sigma-Aldrich, DUO92005), PLA probe anti-mouse plus (Sigma-Aldrich, 92001) and Duolink™ In Situ Detection Reagents Red (Sigma-Aldrich, DUO92008). The assay produces a signal when the distance between the PLA probes is less than 40 nm.

### Reverse Transcription PCR

Total RNA was extracted from organotypic cerebellar slices harvested at DIV 5-7 and cDNA was synthesized with reverse transcription PCR using oligo(dT) primers (Applied Biosystems, Foster City, USA). For gene expression analysis, RT-qPCR reactions were conducted in a total volume of 20 μl comprising 10 μl of Mastermix with SYBR green (Applied Biosystems, Foster City, USA), 0.5 μl of each primer (1.0 μM), 0.3 μl of sample cDNA and 8.5 μl ultrapure water. Real-time PCR reactions were run on a real-time PCR system (Applied Biosystems, Foster City, USA) under the following reaction conditions: 1 cycle of [95 °C for 10 min], 40 cycles of [94 °C for 15 s → 65 °C for 60 s] and 1 cycle of [95 °C for 15 s → 72 °C for 30 s → 95 °C for 15 s]. Oligonucleotide primers were designed using the Primer3 software (http://bioinfo.ut.ee/primer3/). Forward primer: 5′- ACG AGC GAT CGT CTT CTG AT -3′. Reverse primer: 5′- GAA GCG AGT ATG CAC GTC AA -3′. Reactions were quantified by relative standard curve system and the cycle threshold (Ct) method using the SDS2.2 software (Applied Biosystems, Foster City, USA). A relative quantitation value (RQ) for each sample from the triplicates of that sample was calculated for each gene. The data were analysed as RQ for the gene of PKCγ transgenic mice/ RQ for control mice.

### Organotypic Cerebellar Slice Cultures

Animal experiments were carried out in accordance with the EU Directive 2010/63/EU for animal experiments and the study design and the procedures were reviewed and approved by Swiss authorities. Organotypic cerebellar slice cultures were prepared as previously described [10]. Briefly, postnatal day 8 mice were decapitated, the cerebellum removed and placed in ice-cold preparation medium (minimal essential medium (MEM) with Glutamax (Life Technologies, Zug, Switzerland), pH 7.3). The cerebellum was cleaned from choroid plexus and cerebral membranes, placed on a McIllwain tissue chopper and cut in sagittal slices of 350 μm thickness. Millicell cell culture inserts (PICM03050, Millipore, Zug, Switzerland) were pre-wet with Neurobasal medium (Neurobasal A medium (Life Technologies, Zug, Switzerland) supplemented with B27 supplement (Life Technologies, Zug, Switzerland) and Glutamax, pH 7.3) and the slices carefully placed on the membrane. Cultures were maintained for 7 days with medium changes every 2–3 days. For treatments with PMA, 1.5 µL of a 50 µM stock solution were added to 750 µL (final concentration = 100 nM) of medium and added to the cultures with the first medium exchange at day 3 or 4 in vitro and at every following medium exchange. 1.5 µL DMSO in 750 µL medium were used in control wells.

### Western Blot Analysis

Lysates were prepared as described. 2x Laemmli buffer was added and samples loaded onto polyacrylamide gels. Gels were run at 120 V for 75 min in running buffer (0.25 M Tris, 1.93 M glycine, 0.1% SDS). Proteins were transferred to nitrocellulose membranes (Bio-Rad Laboratories, Cressier, Switzerland) in transfer buffer (0.25 M Tris, 1.93 M glycine, 20% methanol) at 350 mA for 45 mins. Membranes were blocked in 5% BSA in TBS for 1 h and incubated with primary antibodies including rabbit anti-CRMP2 (1:1000; Sigma-Aldrich; C2993), rabbit anti-PKCγ (1:1000; Santa Cruz Biotechnology, Santa Cruz, USA; sc-211), rabbit anti-pCRMP2 (1:500; ECM Biosciences; CP2251) and mouse anti-actin (1:2000; Sigma-Aldrich; A5441) overnight at 4 °C. Secondary antibodies IRDye® 800CW Donkey anti-mouse (Li-Cor, Bad-Homburg, Germany; 926-32212) and IRDye® 680LT Donkey anti-rabbit (Li-Cor; 926-68023) were added at a concentration of 1:5000 diluted in TBS-T. After washing, the signal was detected using the Odyssey® Fc Imaging System and software (LI-COR Biosciences, Bad Homburg, Germany). To determine the ratios, pCRMP2/CRMP2 gels were run in parallel for total CRMP2 and pCRMP2. The immunoreactivity in each gel was normalized to the actin signal and the ratio evaluated by (pCRMP2/actin)/(CRMP2/actin) and then normalized to wild-type controls.

### Nucleofection of Dissociated Cerebellar Cultures

Preparation of dissociated cultures was performed as previously described [26, 27]. Briefly, mice were sacrificed by decapitation at postnatal day zero (p0). The cerebellum was removed from the skull, placed in ice-cold Hanks’ balanced salt solution (HBSS, Sigma-Aldrich, Buchs, Switzerland) and cut into 1 mm^2^ pieces. Three hundred microliters of papain solution (HBSS containing 20 U/ml papain (Worthington, Lakewood, USA) was added and the mixture was incubated at 37 °C for 20 min. Digestion was arrested by addition of 500 μL HBSS (including 5% FBS, GIBCO, Invitrogen) and the solution was spun down at 700 rpm for 4 min. The pellet was then dispersed in 350 μL DNAse1 solution (0.02% DNase1, 11.88 mM MgSO_4_, 1 mL HBSS) and passed through a 180 μm nylon net filter (Millipore, Zug, Switzerland). The cell solution was spun down and the pellet was washed in 1 mL HBSS. After centrifugation, the pellet was dissociated in 85 μL transfection solution and 5 μL DNA (1 μg/μL) was added. The cell-DNA mixture was then transferred to Lonza cuvettes and transfected using the O-0003 program of the Nucleofector™ 2b. Immediately after transfection, 200 μL of dissociated culture medium (DMEM/F-12, 1x Glutamax, 1x N2-supplement (Life Technologies, Zug, Switzerland) 100 nM tri-iodothyronine (Merck, Darmstadt, Germany)) supplemented with 10% FBS was added to the cells and the suspension was transferred to 8-well chamber-slides. The cultures were maintained for 14–16 days and the medium changed every 4–5 days.

### Plasmid Construction

Collapsin response mediator protein 2 (CRMP2) is encoded by the *DPYSL2* gene. For simplification, we apply the most commonly used name, CRMP2, in this manuscript.

pCMV6-Dpysl2 (NM_009955) Mouse Untagged Clone (Origene, Rockville, USA; MC205233) was used as a template and the CRMP2 coding sequence was amplified using the following primers: CRMP2-ORF-pL7-F: 5′-GTC CGG ACT CAG ATC TAT GTC TTA TCA GGG GAA GAA AAA T-3′; CRMP2-ORF-pL7-R: 5′-AGC AGG ATC CGT CGA CTT AGC CCA GGC TGG TGA TGT-3′. The pL7-GFP vector [26] was linearized for 1 h at 37 °C in the appropriate buffer using BglII (New England BioLabs, Ipswich, USA; R0144S) and SalI-HF (New England BioLabs, Ipswich, USA; R3138S) restriction enzymes. The linearized vector and PCR product were then fused using the In-Fusion® HD Cloning kit (Clontech, Mountain View, USA; 638912).

### Site-Directed Mutagenesis

To insert the T555A and T555D mutation, the Muta GeneArt™ Site-Directed Mutagenesis system was used (Life Technologies, Carlsbad, USA) with the following primers and the pCMV6-Dpysl2 plasmid as template: T555A-F: 5′-ATT CCC CGC CGC ACC GCC CAG CGC ATC GTG GCA-3′; T555A-R: 5′-TGC CAC GAT GCG CTG GGC GGT GCG GCG GGG AAT-3′; T555D-F: 5′-ATT CCC CGC CGC ACC GAC CAG CGC ATC GTG GCA-3′; T555D-R: 5′-TGC CAC GAT GCG CTG GTC GGT GCG GCG GGG AAT-3′. In short, the pCMV6-Dpysl2 plasmid was methylated and amplified using the aforementioned primers. The original plasmid was then removed in an enzymatic reaction using McrBC endonuclease. The products were transformed into DH5α™-T1R E.Coli (included in kit). Colonies were picked and analysed by Ecoli NightSeq (Microsynth, Balgach, Switzerland). The sequences including the respective mutations were then subcloned into the pL7-GFP vector as described.

### miRNA-Mediated Knockdown of CRMP2

miRNA knockdown constructs were created using the BLOCK-iT™ Pol II miR RNAi Expression Vector Kit (Thermo Fisher Scientific, Rockford, USA). Pre-miRNAs were designed using the BLOCK-iT™ RNAi Designer and cloned into the BLOCK-iT™ Pol II miR RNAi expression vector according to the manufacturer’s protocols. Briefly, top- and bottom-strand oligos were annealed and the double-stranded product was cloned into the linearized pcDNA™6.2-GW/miR vector. The ligation reaction was transformed into Stellar competent cells (Clontech, Mountainview, USA) and the bacteria plated onto agarose plates containing 50 μg/mL spectinomycin. Colonies were analysed using Ecoli NightSeq (Microsynth, Balgach, Switzerland). Vectors were then sequenced and tested for their efficacy by transfection into HEK293T cells (RRID: CVCL_0063; ATCC; Molsheim Cedex, France) using the Effectene transfection reagent (Qiagen, Hilden, Germany). The most effective sequence was subcloned into a pL7- vector containing a His-GST-fusion protein to validate miRNA expression after transfection. Transfections were performed as mentioned.

### Generation of CRMP2-T555A Knock-In Mice

Animal experiments were carried out in accordance with the EU Directive 2010/63/EU for animal experiments and the study design and the procedures were reviewed and approved by Swiss authorities. Knock-in mice with the single point mutation (c. 1663 A > G; p. Thr555Ala) in the *DPYSL2* gene (Chromosome 14, 34.60 cM) were generated in a FVB background using the CRISPR/Cas9 engineering system at the Center of Transgenic Models, University of Basel, using the rapid oocyte injection method. Alt-R® CRISPR-Cas9 crRNA (5′-GGG TGC CAC GAT GCG CTG GGT GG-3′), Alt-R® CRISPR-Cas9 tracrRNA and Donor DNA (5′-CAC TCG TTT CTT GTC TCT TCT TTC TTC TCT AGG TGC TCA GAT TGA CGA CAA CAT TCC CCG CCG CAC AGC TCA GCG CAT CGT GGC ACC CCC TGG TGG CCG TGC CAA CAT CAC CAG CCT GGG CTA AAG CCC CTA GGC CTG CAG GCC ACT TGG GGA TGG GGG ATG GGA CAC CTG AGG ACA TTC TGA GAC TTC CTT TCT TCC AT-3′) were obtained from Integrated DNA Technologies (USA). CRISPR RNA, Cas9 and Donor DNA were injected into FVB zygotes at the pronuclear site and surviving embryos were transferred into pseudopregnant mice. To identify founders, genotyping with genomic DNA samples from biopsies was performed by PCR. The primers for genotyping were forward, 5′- AGG ATT GTT CCT GGG CAT AC -3′ and reverse 5′- TCA TGA ACA CCA CAC CCA AG -3′. Then, the point mutation at 1663 A > G *DPYSL2* gene was confirmed by DNA sequencing. The fragment for sequencing was obtained by PCR with genomic DNA samples and primers as mention. The confirmed point mutation knock-in founders were crossed with FVB mice (Jackson Laboratory, Sacramento, USA) to generate heterozygous CRMP2-T555A mice. These were crossed with other heterozygous CRMP2-T555A mice to obtain wild-type, heterozygous and homozygous CRMP2-T555A knock-in mice.

### Immunohistochemistry of Cerebellar Slice Cultures and Cerebellar sections

Mice were euthanized by an overdose of Pentobarbital and perfused with 4% PFA in PBS + 5% sucrose. The brain was dissected, postfixed overnight and cryoprotected in 30% sucrose for one day. Brains were mounted in Tissue-Tek (Tissue-Tek® O.C.T.™ Compound, Sakura, Ca. no. SA62550-01) and frozen by immersion in –40 °C isopentane. Sagittal sections were cut at 25 µm on a Leica CM1900 cryostat. Cultures were fixed in 4% PFA and then washed three times with phosphate buffer (PB). Primary antibodies (mouse anti-Calbindin, 1:500, Swant, Marly, Switzerland; No. 300; rabbit anti-CRMP2, 1:1000; Sigma-Aldrich, St. Louis, USA, No. C2993 and rabbit anti-pCRMP2, 1:200; ECM Biosciences; No. CP2251) were diluted in PB with 0.03% Triton-X and 3% normal goat serum (GIBCO, Invitrogen). Slices or sections were then incubated free-floating overnight at 4 °C under gentle agitation. After three washes with PB, secondary antibodies Alexa Fluor-568 goat anti-rabbit (Molecular Probes, Eugene, USA; A11011) and Alexa Fluor-488 goat anti-mouse (Molecular Probes, Eugene, USA; A11001) were added at a concentration of 1:500 in PB with 0.01% Triton-X. Sections or slices were washed again three times for 5 min and then mounted in Mowiol. Sections were viewed on an Olympus AX-70 microscope equipped with a Spot digital camera. For slice cultures, confocal microscopy was performed on an upright laser scanning microscope (Zeiss LSM700) equipped with solid-state lasers. Images were acquired using a Plan-Apochromat 40x/1.3 Oil DIC M27 objective (Zeiss) and standard PMT detectors. Images were adjusted for contrast and brightness.

### Immunohistochemistry of Dissociated Cultures

Slides were fixed in 4% PFA and then washed 3x with phosphate buffer (PB). Primary antibodies including mouse anti-Calbindin (1:500; Swant, Marly, Switzerland; 300), rabbit anti-Calbindin (1:1000; Swant, Marly, Switzerland; CB38), rabbit anti-GFP (1:500; Abcam, Cambridge, UK; ab6556) and mouse anti-His (1:200; Life Technologies, Carlsbad, USA; 710,286) were diluted in PB with 0.03% Triton-X and 3% normal goat serum and slides were incubated for 1 h at RT. Slides were washed three times with PB and secondary antibodies Alexa Fluor-568 goat anti-rabbit (Molecular Probes, Eugene, USA; A11011), Alexa Fluor-488 goat anti-mouse (Molecular Probes, Eugene, USA; A11001), Alexa Fluor-568 goat anti-mouse (Molecular Probes, Eugene, USA; A11004) and Alexa Fluor-488 goat anti-rabbit (Molecular Probes, Eugene, USA; A11008) were added at a concentration of 1:500 in PB with 0.01% Triton-X. Slides were washed three times, the wells were detached from the glass and the slide mounted in Mowiol. Cultures were viewed on an Olympus AX-70 microscope equipped with a Spot digital camera. Images were adjusted for contrast and brightness.

### Statistical Analysis

The quantification of Purkinje cell dendritic tree size from organotypic slice cultures or dissociated Purkinje cell cultures was done as previously described [10]. Only Purkinje cells not overlapping with other cells were chosen for the analysis. Dendritic area was measured by outlining each cell in ImageJ. A minimum number of 40 cells per group from at least three independent experiments were included in the analysis. In case of transfection of CRMP2^ki/ki^ dissociated cultures, two independent experiments were analysed. GraphPad Prism was used for statistical analyses using the non-parametric Mann-Whitney’s test. Statistical significance was assumed when *p* < 0.05.

## Results

### CRMP2 Interacts with PKCγ in the Cerebellum

Cerebella from p12 PKCγ(S361G)-mice or PKCγ^−/−^-mice were lysed and PKCγ was immunoprecipitated using protein A/G coated agarose beads. Proteins pulled down along with PKCγ were identified using a shotgun mass spectrometry approach. Beads prepared with anti-vGlut1 antibody, a glutamate transporter expressed in axon terminals of cerebellar granule cells, were included in the experiment as a negative control. To exclude falsely identified, non-specifically bound proteins, the same experiment was performed using cerebellar lysates prepared from PKCγ^−/−^-mice. Amongst the proteins co-purified from PKCγ(S361G)-lysates, we identified CRMP2. Total identified spectra for CRMP2 were normalized to the total amount of spectra identified in each sample. The number of normalized total spectra counts for CRMP2 was strongly decreased in the PKCγ^−/−^-pulldowns, confirming the specific binding of CRMP2 to PKCγ (Table 1).

**Table 1:** Mass spectrometry analysis of cerebellar lysates from PKCγ(S361G)- and PKCγ^−/−^-mice. Numbers indicate normalized total spectra of two sample duplicates identified for the proteins indicated above (see supplementary Table 1 for a detailed report). The fold changes of PKCγ and CRMP2 detected in PKCγ-precipitated samples are shown

Bait	PKCγ (sp|P63318|)		CRMP2 (sp|O08553|)	
	PKCγ(S361G)	PKCγ^−/−^	PKCγ(S361G)	PKCγ^−/−^
vGlut1	1 | 1	0 | 0	5 | 5	4 | 1
PKCγ	33 | 34	0 | 0	28 | 35	8 | 4
CRMP2	1 | 0	0 | 0	32 | 31	27 | 30
Fold change (PKCγ(S361G)/ PKCγ^−/−^)	0.0		0.2	
*p* value (Student’s *t* test)	< 0.00010		0.027	

**Fig. 1:**
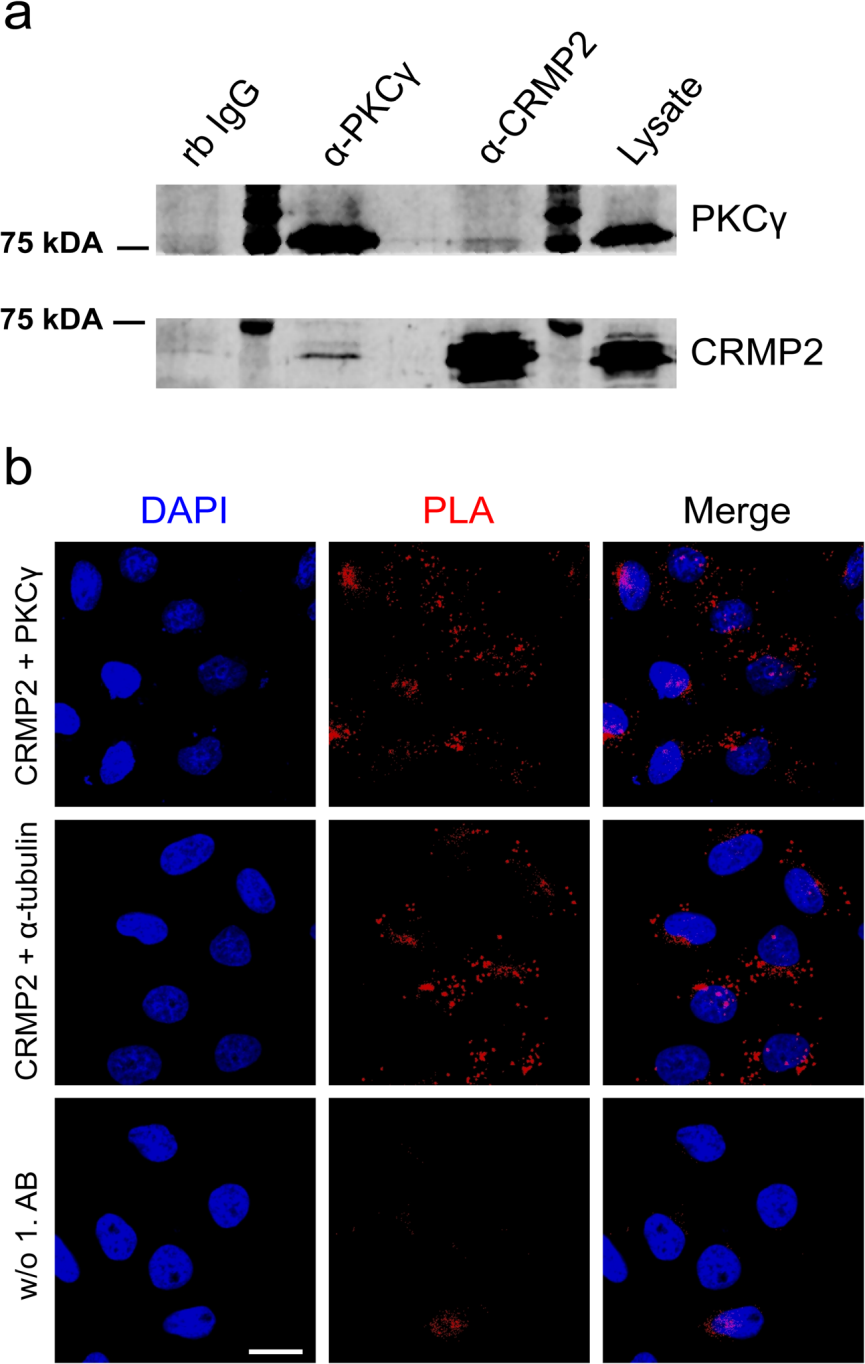
Interaction of CRMP2 and PKCγ. **a** Immunoblot showing the co-immunoprecipitation of CRMP2 and PKCγ from cerebellar lysates. Proteins were detected with the indicated antibodies. Normal rabbit IgG was used as a negative control. **b** HeLa cells were probed with α-PKCγ and α-CRMP2 antibody and the interaction detected via Duolink proximity ligation assay (top row); α-CRMP2 and α-alpha-tubulin antibodies were used as a positive control (middle row) and primary antibodies were omitted as a negative control (bottom row). Blue, nuclei stained with DAPI; red, PLA signal. Scale bar = 20 μm

In PKCγ-immunoprecipitations, CRMP2 was detected along with PKCγ in immunoblots, which was not the case when control rabbit IgG was used in the precipitation. The same was true in precipitations of CRMP2, where PKCγ and CRMP2 were both identified (Fig. 1a).

In order to confirm the interaction, we used the Duolink™ proximity ligation assay in HeLa cells (Fig. 1b). As a positive control of a known CRMP2 interaction, we performed the assay using primary antibodies against CRMP2 and alpha-tubulin, both natively expressed in HeLa cells. After the addition of the Duolink plus and minus probes, this reaction produced a positive signal. When primary antibodies were omitted, the PLA signal could be abolished. HeLa cells transfected with wild-type PKCγ and labelled with antibodies against CRMP2 and PKCγ also displayed the PLA signal, verifying the close association of the two proteins.

### Phosphorylation of CRMP2 Is Increased in PKCγ(S361G)-Mice

**Fig. 2:**
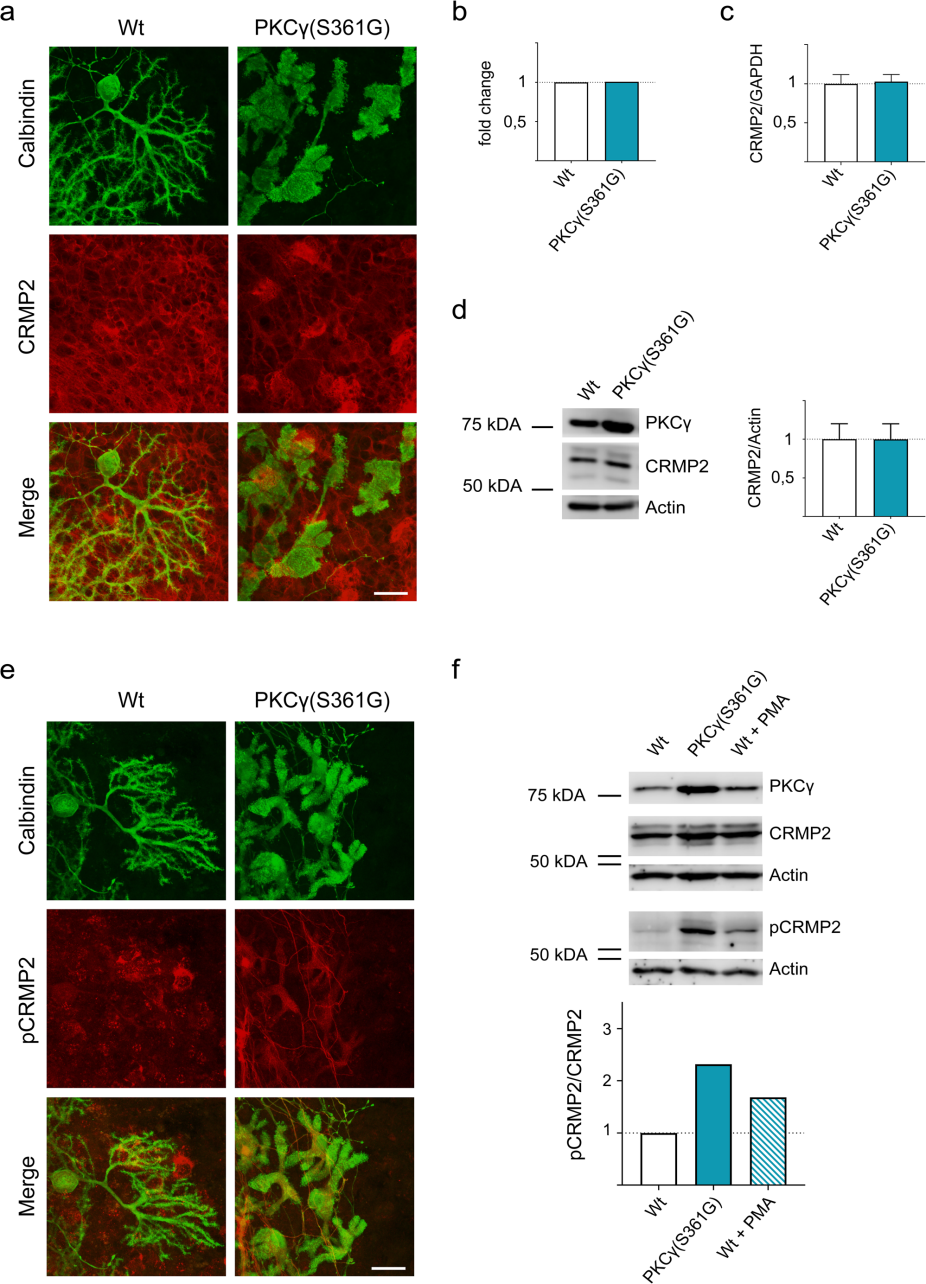
Phosphorylation of CRMP2 is increased in PKCγ(S361G)-mice. **a** Immunostainings of CRMP2 in cerebellar OTSCs from Wt- and PKCγ(S361G)-mice show no visible differences in localization or expression of CRMP2. **b** No changes in gene expression were detected in a gene chip assay and **c** quantitative RT–PCR. **d** Immunoblot of CRMP2 expression in cerebellar lysates prepared from Wt- and PKCγ(S361G)-mice. Summary of the Western blot analysis of five independent experiments showing that the expression of CRMP2 normalized to actin is unchanged. **e** Immunostainings of pCRMP2 in cerebellar OTSCs from Wt- and PKCγ(S361G)-mice show an upregulation of phosphorylated CRMP2 in Purkinje cells. **f** Immunoblot of cerebellar lysates prepared from OTSCs. There is an increase of pCRMP2 in PKCγ(S361G)-OTSCs or Wt-cultures treated with 1 μM PMA. Summary of the Western blot analysis showing the mean of five independent experiments. The expression of pCRMP2 normalized to total CRMP2 is upregulated approximately 2.3-fold in PKCγ(S361G)-mice and 1.7-fold in PMA treated cultures compared to untreated controls. Scale bars = 25 μm

In order to characterize the relation of CRMP2 to PKCγ, we used organotypic slice cultures (OTSCs) prepared from wild-type and PKCγ(S361G)-mice. Immunostainings confirmed that CRMP2 is expressed in various cell types of the cerebellum and that there was no apparent difference in localization or expression of the protein between wild-type and PKCγ(S361G)-mice (Fig. 2a). We further assessed the gene and protein expression of CRMP2 in PKCγ(S361G)-mice. When we analysed data from a gene chip microarray [27], we found no significant differences in CRMP2 mRNA expression between wild-type- and PKCγ(S361G)-mice (Fig. 2b). RT-qPCR confirmed that mRNA expression levels were unaltered (Fig. 2c). We then used lysates of OTSCs prepared from PKCγ(S361G)-mice for Western blotting and detected no differences in CRMP2 expression in PKCγ(S361G)-mice (Fig. 2d).

Since CRMP2 functions are primarily regulated by posttranslational modifications, we decided to focus on the Thr555 phosphorylation site, which had been previously shown to be a target of PKC [21]. In PKCγ(S361G)-transgenic mice, while total CRMP2 expression and localization remained unchanged, levels of CRMP2 phosphorylated at Thr555 (pCRMP2) were strongly increased in OTSCs. The upregulation of pCRMP2 in PKCγ(S361G)-mice was essentially confined to Purkinje cells confirming the strong cell-specific upregulation (Fig. 2e). Analysis of immunoblots comparing samples from organotypic slice cultures also confirmed the increase in CRMP2 phosphorylation in PKCγ(S361G)-cultures and in wild-type cultures treated with the PKC activator Phorbol 12-myristate 13-acetate (PMA) (Fig. 2f). It therefore seems likely that the increase in CRMP2 phosphorylation is a consequence of increased PKCγ kinase activity in PKCγ(S361G)-mice while overall expression of CRMP2 remained unaffected.

### Knockdown of CRMP2 Impairs Dendritic Development of Cerebellar Purkinje Cells

**Fig. 3:**
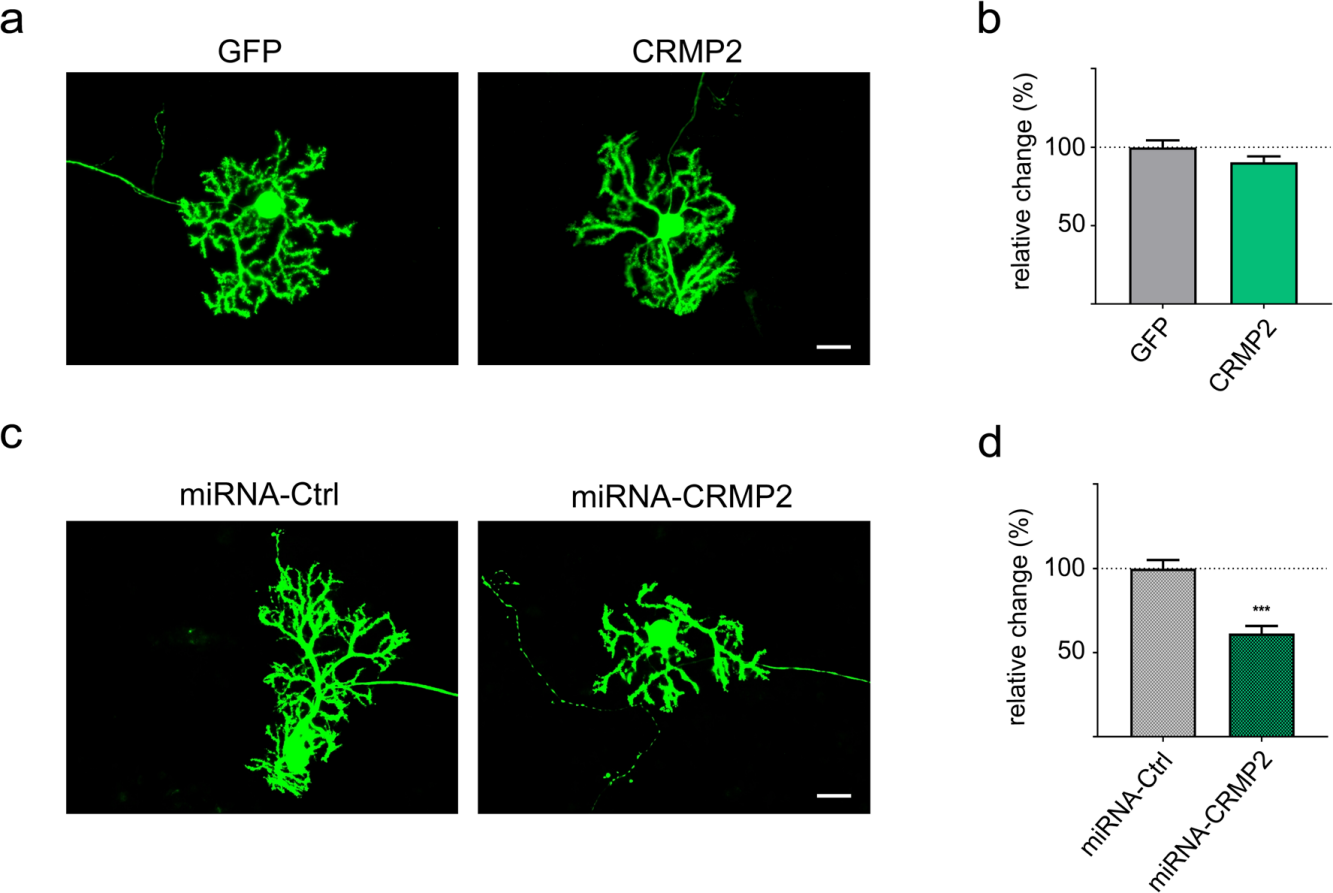
CRMP2 regulates dendritic development of cerebellar Purkinje cells. **a** Dissociated cerebellar cultures showing Purkinje cells stained with calbindin. Cultures were transfected with GFP or GFP-tagged wild-type CRMP2 under the control of a Purkinje cell-specific promotor to overexpress wild-type CRMP2. More examples of Purkinje cells for the different treatments are shown in supplementary Fig. 3. **b** Quantification of Purkinje cell dendritic area shows no significant changes (GFP only: *n* = 49; CRMP2-GFP: *n* = 109). **c** miRNA-mediated knockdown of CRMP2. Transfection of dissociated cultures using knockdown constructs containing the L7-promotor, a His-GST reporter and either a control miRNA or CRMP2 specific miRNA. **d** Quantification of the gross dendritic area shows a statistically significant decrease of the Purkinje cell dendritic area after CRMP2 knockdown (*p* < 0.001; miRNA-Ctrl: *n* = 130; miRNA-CRMP2: *n* = 73); Data were acquired from 4 independent experiments respectively and are shown as the mean ± SEM. Scale bar = 25 μm

Since phosphorylation of CRMP2 was upregulated specifically in cerebellar Purkinje cells in PKCγ(S361G)-mice, we aimed to further investigate the role of CRMP2 for dendritic development of Purkinje cells. We first overexpressed wild-type CRMP2 (Wt-CRMP2) with an added GFP-tag in dissociated cerebellar cultures specifically in Purkinje cells using a vector containing the L7-promotor. Purkinje cells transfected with a GFP control vector showed normal dendritic development. Also, the overexpression of CRMP2 did not alter dendritic development as determined by measuring the Purkinje cell dendritic area (Fig. 3a, b).

We then explored the effect of a reduction of CRMP2 by miRNA-mediated knockdown of the protein. The miRNA sequence used for transfection was selected based on its knockdown efficiency in HEK293T cells (Supplementary Fig. 1 a). The miRNA was then expressed using a L7-plasmid containing a His-GST fusion protein as a reporter. Purkinje cells transfected with the miRNA displayed reduced CRMP2 expression levels (Supplementary Fig. 1 b) and had a significantly decreased Purkinje cell dendritic area (Fig. 3c, d). This finding shows that CRMP2 is required for correct dendritic development in Purkinje cells while an excess of the wild-type protein does not seem to exert a negative effect.

### Purkinje Cell Dendritic Development Is Modulated by CRMP2 Phosphorylation

**Fig. 4:**
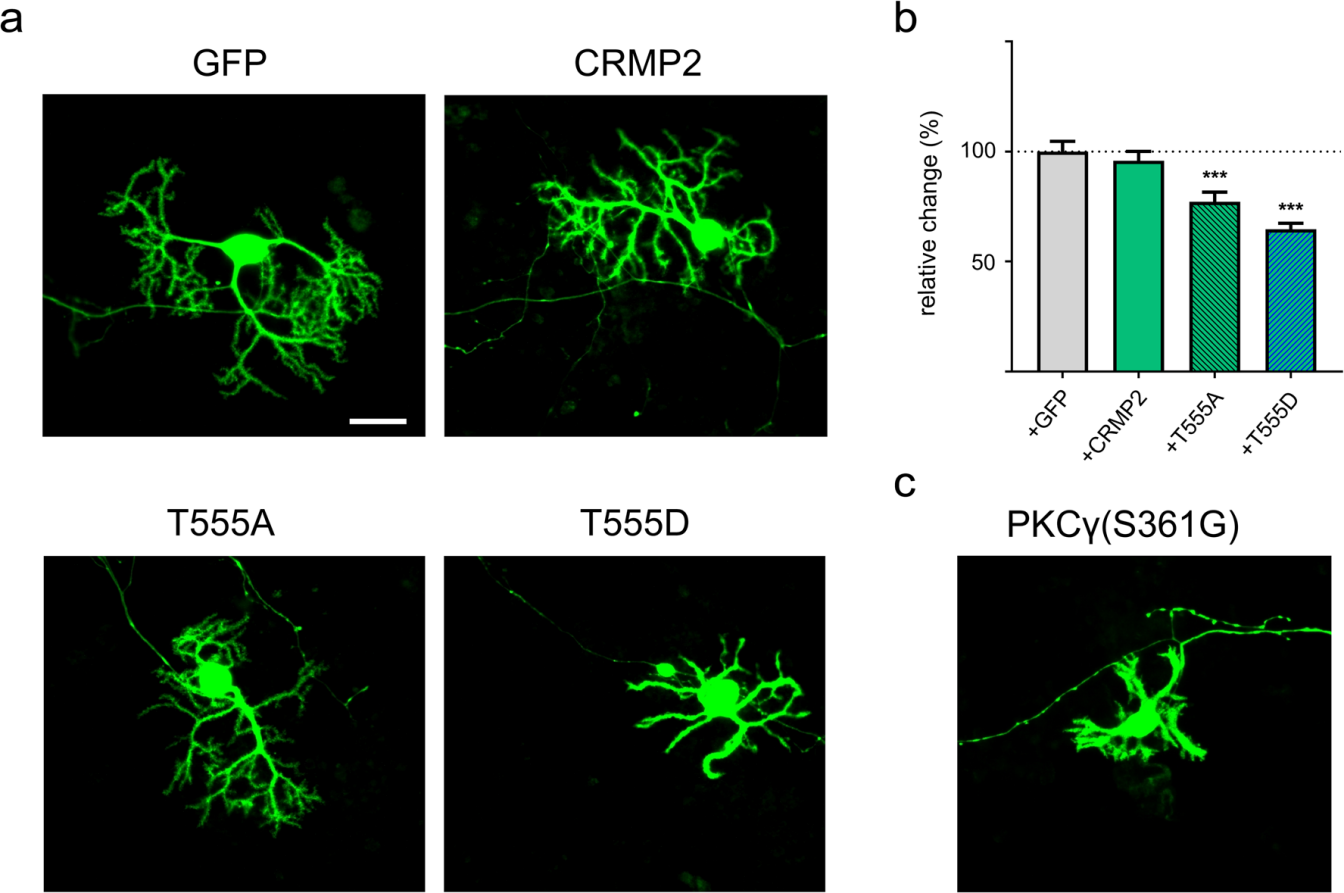
Dendritic development in Purkinje cells is modulated by CRMP2 phosphorylation. **a** Dissociated cerebellar cultures showing Purkinje cells expressing GFP. Cells were transfected with GFP or GFP-tagged wild-type CRMP2, phospho-defective (T555A) or phospho-mimetic (T555D) CRMP2 under a Purkinje cell-specific promotor. More examples of Purkinje cells for the different treatments are shown in supplementary Fig. 4. Scale bar = 25 μm. **b** Analysis of the Purkinje cell dendritic area shows a statistically significant reduction in cells transfected with T555A- or T555D-CRMP2 (*p* < 0.001; GFP only: *n* = 49; CRMP2-GFP: *n* = 109; T555A-GFP: *n* = 58; T555D: *n* = 126). **c** Example of a typical Purkinje cell in dissociated cerebellar cultures prepared from PKCγ(S361G) mice showing the typical stunted dendritic morphology. Data were acquired from 4 independent experiments of GFP-, CRMP2- and T555D-transfections and 3 independent experiments for T555A. Data are shown as the mean ± SEM

Since Purkinje cell dendritic development is impaired in PKCγ(S361G)-mice, we investigated the relationship between the phosphorylation of CRMP2 and dendritic outgrowth and created constructs for the expression of phospho-defective (T555A) and phospho-mimetic (T555D) mutants of CRMP2. The mutant-CRMP2 variants were expressed with a GFP-tag under the control of the L7-promotor for Purkinje cell-specific expression and transfected into dissociated cerebellar cultures. Transfection of the phospho-defective T555A-CRMP2 resulted in a small but significant decrease of Purkinje cell dendritic area (Fig. 4a, b). Transfection of the phospho-mimetic T555D mutant resulted in a more pronounced reduction of the Purkinje cell dendritic area (Fig. 4a, b). Since transfected Purkinje cells still express native CRMP2 that retains the ability to be phosphorylated, potential effects of transfected T555A-CRMP2 could have been quenched by native CRMP2. The phospho-mimetic mutant resulted in a strong inhibition of dendritic growth reminiscent of the morphology seen in Purkinje cells derived from PKCγ(S361G)-mice (Fig. 4c). As CRMP2-phosphorylation is greatly increased in PKCγ(S361G) transgenic Purkinje cells, the phosphorylation of CRMP2 could be one of the mediators of the reduction of dendritic growth seen in these cells.

### Generation of CRMP2^ki/ki^-Mice

**Fig. 5:**
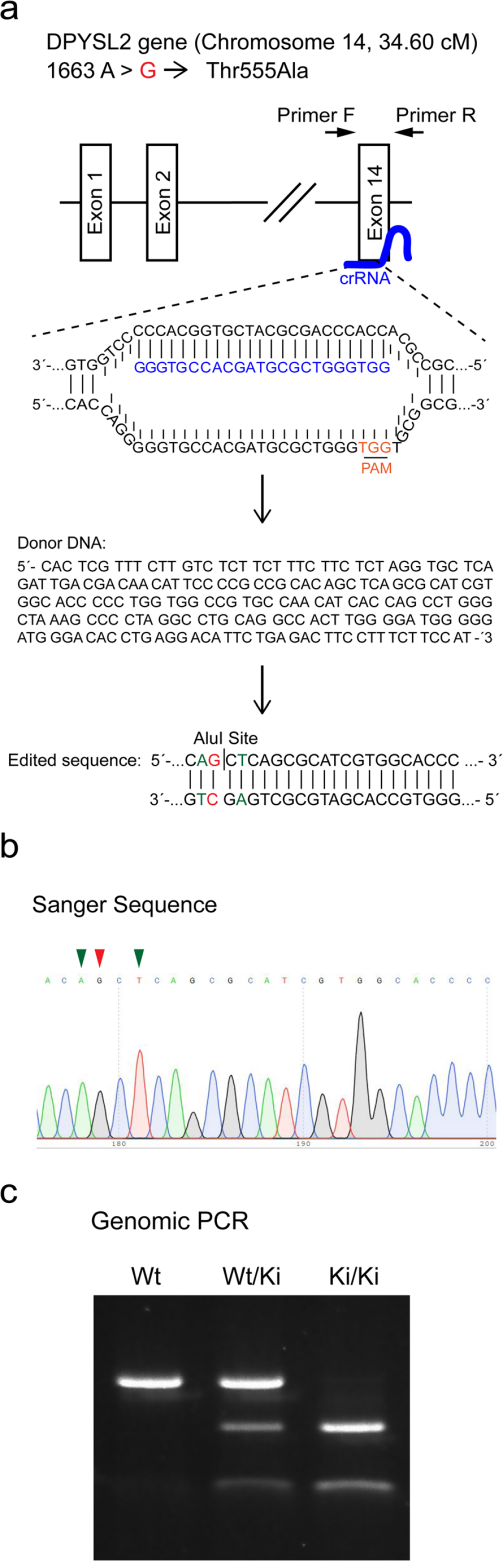
Generation of CRMP2^ki/ki^-mice. The single point mutant (c. 1663 A > G; p. Thr555Ala) in knock-in mice was generated in FVB background with the CRISPR/Cas9 engineering system. **a** Schematic representation of the *DPYSL2* gene (Chromosome 14, 34.60 cM) and the CRISPR RNA target locus. crRNA sequence is outlined in blue. The protospacer adjacent motif (PAM) is outlined in orange. The displayed donor DNA sequence was introduced to generate the edited final sequence including an AluI restriction site. The 1663 A > G mutation is indicated in red and mutations of the restriction site in green. **b** The mutations were confirmed by DNA sequencing. Arrowheads successful introduction of the respective mutations. **c** Genomic PCR shows multiple bands after AluI digestion, indicating presence of the mutation

**Fig. 6:**
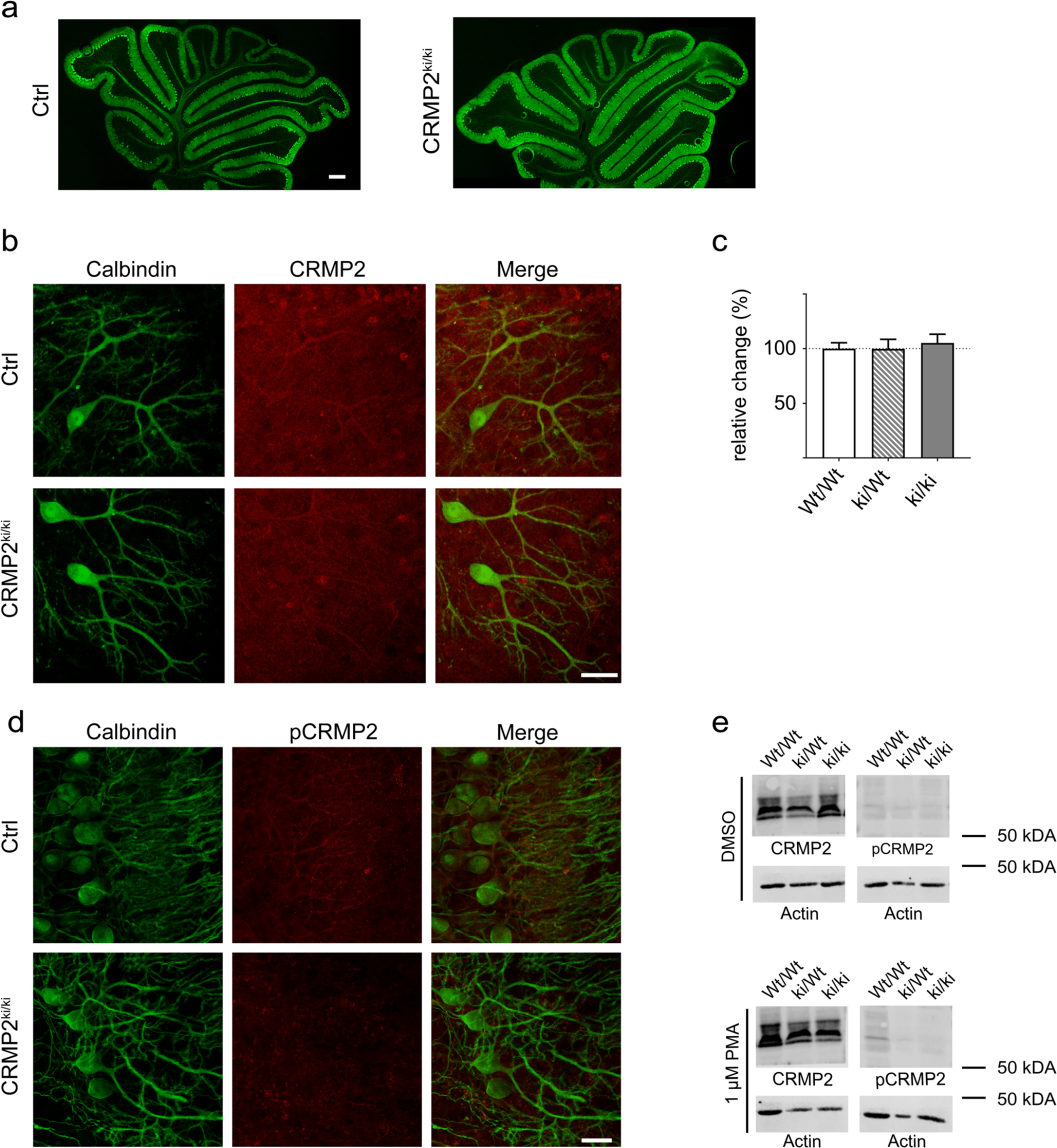
CRMP2 phosphorylation at Thr555 is abolished in CRMP2^ki/ki^-mice. **a** Frozen cerebellar sections from Ctrl- and CRMP2^ki/ki^-mice (postnatal day 12); Scale bar = 200 μM. Gross cerebellar morphology appears normal in CRMP2^ki/ki^-mice. **b** Immunostainings of OTSCs prepared from from Ctrl- and CRMP2^ki/ki^-mice show no differences in CRMP2 expression. Scale bar = 25 μm; **c** Cerebellar OTSCs from CRMP2^ki/ki^-mice show normal development as shown by the analysis of Purkinje cell dendritic area (CRMP2^wt/wt^: *n* = 93; CRMP2^ki/wt:^ *n* = 63; CRMP2^ki/ki^: *n* = 77). Data were acquired from 4 independent experiments of CRMP2^wt/wt^-cultures and 3 independent experiments for CRMP2^ki/wt^ for CRMP2^ki/ki^. Data are shown as the mean ± SEM. **d** Immunostainings of pCRMP2 in cerebellar OTSCs from Ctrl- and CRMP2^ki/ki^-mice show that CRMP2 phosphorylation was successfully abolished; Scale bar = 25 μm; **e** Immunoblots of CRMP2 and pCRMP2 expression in cerebellar OTSCs from CRMP2^wt/wt^-, CRMP2^ki/wt^- and CRMP2^ki/ki^-mice show that pCRMP2 expression is abolished in knock-in animals and cannot be increased by treatment with PMA

Because the presence of native CRMP2 could have compensatory effects in transfection experiments, we created a CRMP2 knock-in mouse carrying the T555A mutation using the CRISPR/Cas9/-system in an FVB background (Fig. 5a–c). In these knock-in mice, general cerebellar development and Purkinje cell dendritic development in OTSCs from CRMP2^ki/ki^-mice was unchanged (Fig. 6a–c). Immunostainings showed that expression levels of CRMP2 remained unchanged in CRMP2^ki/ki^-OTSCs compared to cultures prepared from control animals (Fig. 6b), while phosphorylation of CRMP2 at Thr555 was abolished (Fig. 6d). When these cultures were treated with PMA, there was no increase in pCRMP2 (Fig. 6e), confirming the successful knock-in of the mutation.

### Dendritic Development in Dissociated Cerebellar Cultures from CRMP2^ki/ki^-Mice Is Impaired and Can Be Rescued by Transfection of Wt- or T555D-CRMP2

**Fig. 7:**
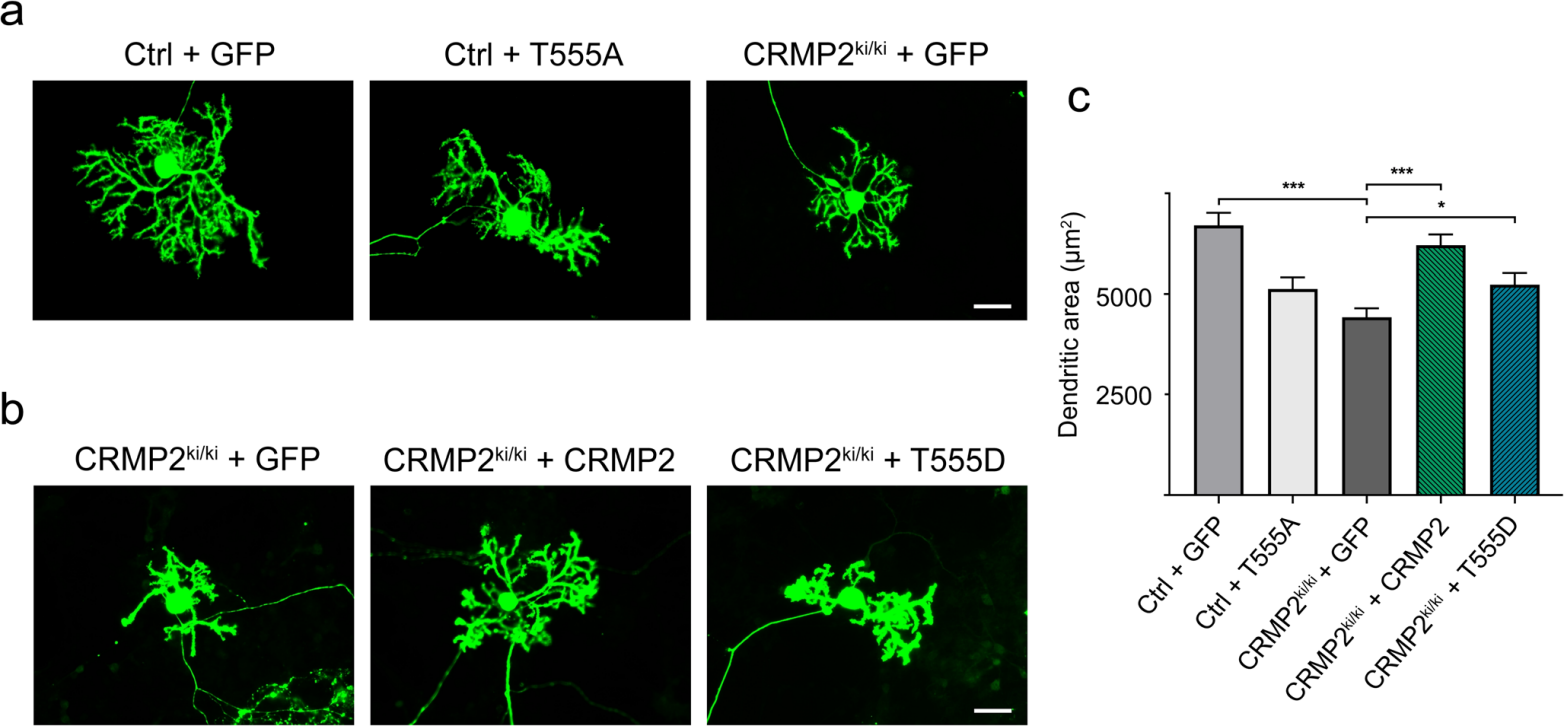
Dendritic development in CRMP2^ki/ki^-mice is impaired in dissociated cerebellar cultures and can be rescued by transfection of Wt- or T555D-CRMP2. **a** Dissociated cerebellar cultures prepared from Ctrl-mice transfected with GFP only or GFP-T555A compared to cultures prepared from CRMP2^ki/ki^-mice transfected with GFP only. **b** Dissociated cultures from from CRMP2^ki/ki^-mice transfected with GFP only, GFP-CRMP2 or GFP-T555D. More examples of Purkinje cells for the different treatments are shown in supplementary Fig. 5. **c** Analysis of Purkinje cell dendritic area shows a statistically significant reduction of Purkinje cell dendritic area in CRMP2^ki/ki^-cells transfected with GFP when compared to controls transfected with GFP (*p* < 0.001). There is a virtually complete rescue effect of the Purkinje cell dendritic area after transfection of Wt-CRMP2 (*p* < 0.001) and a less pronounced rescue after transfection with T555D-CRMP2 (*p* < 0.05) (Ctrl + GFP: *n* = 49; Ctrl + T555A: *n* = 58; CRMP2^ki/ki^ + GFP: *n* = 40; CRMP2^ki/ki^ + CRMP2: *n* = 49; CRMP2^ki/ki^ + T555D: *n* = 40). Data were acquired from 4 independent experiments for Ctrl + GFP, 3 independent experiments for Ctrl + T555A and CRMP2^ki/ki^ + GFP and 2 independent experiments for CRMP2^ki/ki^ + CRMP2 and CRMP2^ki/ki^ + T555D. Data are shown as the mean ± SEM. Scale bars = 25 μm

In contrast to our findings in OTSCs, we observed that in dissociated cerebellar cultures, dendritic development of Purkinje cells derived from CRMP2^ki/ki^-mice was strongly impaired (Fig. 7a-c). The dendritic reduction in this case was more pronounced than when dissociated cultures from control animals were transfected with the T555A-mutant (Fig. 7c). We attempted to rescue CRMP2 function by transfecting the wild-type or the T555D-mutant of CRMP2 into dissociated cerebellar cultures from CRMP2^ki/ki^-mice to reconstitute internal levels of pCRMP2. Purkinje cells transfected with wild-type CRMP2 showed an almost complete rescue that was statistically significant (Fig. 7c). Transfection of the phospho-mimetic CRMP2 only resulted in a much smaller increase of the Purkinje cell dendritic area, which still reached statistical significance (Fig. 7c). These results indicate that dynamic phosphorylation and dephosphorylation of CRMP2 is essential for Purkinje cell dendritic development. This could only be fully restored in the mutant by transfection of Wt-CRMP2; transfection of the phospho-mimetic mutant had a much smaller rescuing effect.

## Discussion

In this manuscript, we have identified CRMP2 as a major target of PKCγ phosphorylation in cerebellar Purkinje cells. The interaction of PKCγ and CRMP2 could be confirmed by co-immunoprecipitation and the proximity ligation assay. Furthermore, CRMP2 phosphorylation at Thr555 was strongly increased in the PKCγ(S361G) mouse model with a constitutive active PKCγ kinase domain. As in this mouse model Purkinje cell development is severely impaired, we have further explored the effects of CRMP2 phosphorylation at Thr555 on dendritic development. Surprisingly, Purkinje cell dendritic development was impaired when CRMP2 expression in Purkinje cells was knocked down or when either a phospho-defective or a phospho-mimetic version of CRMP2, but not when wild-type CRMP2 was overexpressed in Purkinje cells highlighting the crucial role of Thr555 phosphorylation for the regulation of dendritic development. This finding was confirmed in Purkinje cells from a novel knock-in mouse model with a CRMP2 containing a phospho-defective Thr555 phosphorylation site. Dendritic development of Purkinje cells from this mouse model was also strongly impaired and could be restored by transfection of wild-type, but less so with phospho-mimetic CRMP2 suggesting that dynamic phosphorylation and dephosphorylation at this site are required for proper Purkinje cell dendritic development. Our findings show that CRMP2 phosphorylation at Thr555 is a major mediator of the regulation of Purkinje cell dendritic growth by PKCγ.

### Identification of CRMP2 as a Phosphorylation Target and an Interactor of PKCγ in Purkinje Cells

CRMP2 is a well-known member of the CRMP family of proteins controlling axonal outgrowth and dendritic development (for review see Quach et al. 2015, Yamashita and Goshima 2012). The activity and function of CRMP2 is strongly determined by its phosphorylation state [28] and one of its phosphorylation sites, Thr555, is subject to phosphorylation by protein kinase C [21]. We have identified CRMP2 as a protein pulled down by immunoprecipitation of PKCγ followed by mass spectrometry. The interaction of CRMP2 with PKCγ was then confirmed with classical co-immunoprecipitation experiments and a proximity ligation assay. In the PKCγ(S361G) mouse model, there is a Purkinje cell-specific expression of a mutated form of PKCγ with a constitutive active kinase domain. Strikingly, in Purkinje cells from these mice, phosphorylation of CRMP2 at Thr555 is strongly increased confirming phosphorylation by PKCγ. At the same time, the gene and protein expression levels of total CRMP2 remained equal compared to wild-type controls.

CRMP2 is known to regulate neurite outgrowth [22, 29] and regulates dendritic development of CA1 pyramidal neurons [30] and cortical pyramidal cells [31]. We therefore focused on CRMP2 as a potential mediator of the effects of PKCγ for Purkinje cell dendritic development.

### CRMP2 Phosphorylation Regulates Dendritic Outgrowth of Cerebellar Purkinje Cells

We have studied the role of CRMP2 for Purkinje cell dendritic development mostly in dissociated cerebellar cultures allowing the transfection of Purkinje cells with the appropriate expression plasmids. In agreement with our findings that there are only minor changes of CRMP2 protein expression in cells of the PKCγ(S361G) mouse, overexpression of wild-type CRMP2 protein had no detectable effects on dendritic development. In contrast, miRNA-mediated knockdown of CRMP2 led to a reduction of dendritic tree development. This is consistent with the notion that a lack of CRMP2 cannot be fully compensated for by changes in its phosphorylation state when the expression levels simply get too low. These experiments suggest that CRMP2 plays an important role for Purkinje cell dendritic growth. In order to address how CRMP2 phosphorylation affects dendritic development, we generated DNA constructs to transfect mutants of CRMP2 into dissociated cultures that either lacked the ability to be phosphorylated (T555A) or mimicked the phosphorylated state of CRMP2 (T555D). Transfection of both phospho-mutants reduced dendritic tree development markedly, with the T555D-mutant having the more pronounced effect. These results point to a negative effect exerted by phosphorylated CRMP2, which is also consistent with the situation in the PKCγ(S361G) mouse model where CRMP2 phosphorylation is increased and dendritic development is compromised. In fact, phosphorylation of CRMP2 at Thr555 has been associated with decreased neurite outgrowth, growth cone collapse and decreased association to microtubules [22]. Furthermore, increased levels of CRMP2 phosphorylated at Thr555 were found in spinal cord neurons from patients with Alzheimer’s disease and active multiple sclerosis lesions [32, 33]. Interestingly, also, the transfection of the phospho-deficient mutant T555A resulted in a reduction of dendritic development. A simple model assuming that T555A promotes and T555D inhibits dendritic growth thus does not apply, rather transfection of any mutant suppressing dynamic phosphorylation and dephosphorylation appears to have a negative effect.

### Importance of Phosphorylation Dynamics for Purkinje Cell Dendritic Growth Confirmed by T555A-CRMP2^ki/ki^-Mice

It is important to bear in mind that in cultures transfected with the phosphorylation-mutants of CRMP2, Purkinje cells still express the wild-type protein. Therefore, potential detrimental effects of phospho-deficient CRMP2 could have been quenched by endogenous CRMP2. For a better understanding of the effects of deactivated CRMP2 phosphorylation, we generated a knock-in mouse model using the CRISPR/Cas9 system, inserting the T555A point mutation. In this mouse model, potential background effects of native CRMP2 are eliminated.

Morphologically, cerebella from CRMP2^ki/ki^-mice appeared normal and organotypic slice cultures prepared from these mice did not show any overt changes in dendritic outgrowth of Purkinje cells. However, Purkinje cells in dissociated cerebellar cultures show impaired dendritic development which was more pronounced than after transfection with T555A or T555D. This phenotype could be rescued almost completely by transfecting wild-type CRMP2. Transfecting the T555D variant gave some improvement but could not completely rescue the phenotype. This indicates that having both CRMP2-T555A and CRMP2-T555D present in the Purkinje cell is better than only having one variant, but in order to achieve full recovery, the dynamic switch between the phosphorylation states is required. This is in agreement with the known growth dynamics of Purkinje cell dendrites which go through stages of elongation and retraction [34], a process which would require a continuous change between phosphorylation and dephosphorylation of CRMP2.

### PKCγ, CRMP2 Phosphorylation and Purkinje Cell Dendritic Development

**Fig. 8:**
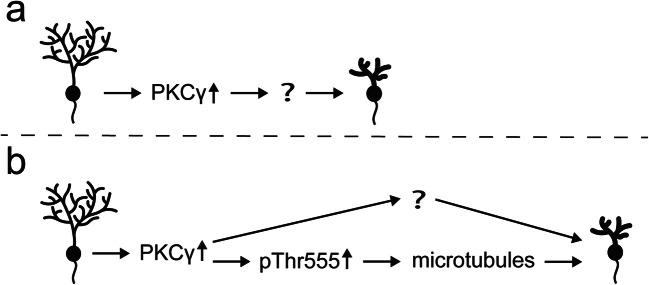
Model for the regulation of Purkinje cell dendritic development via PKCγ activity and CRMP2 phosphorylation. **a** Increased PKCγ activity leads to impaired dendritic development in cerebellar Purkinje cells. The mediators of this effect are largely unknown. **b** We have found that an increase in PKCγ activity results in elevated CRMP2 phosphorylation at threonine 555, which could decrease microtubule stability and impair dendritic development. This can explain some of the effects of increased PKCγ activity on dendritic development

Our findings are compatible with a simple model in which increased activity of PKCγ will lead to increased phosphorylation of CRMP2 at Thr555 and to a destabilization of the dendrites, probably through a destabilization of the microtubule network [29, 35]. CRMP2 in this model is an important downstream effector of increased PKC activity explaining at least partially the impaired dendritic growth seen with PKC activation in Purkinje cells. Dynamic microtubule stability is important for correct dendritic development and knockdown or overexpression of the microtubule destabilizer stathmin both decrease dendritic development of cultured cerebellar Purkinje cells [36]. CRMP2 as a microtubule stabilizing protein could similarly influence microtubule dynamics in Purkinje cell dendritic development (see Fig. 8).

In our experiments, the effects of CRMP2 for dendritic outgrowth were much more evident in dissociated cerebellar cultures compared to the in situ native development of frozen cerebellar sections. This might be explained by the fact that CRMP2 is a member of a family of proteins comprised of CRMP1–5, some of which share similar functions and can interact with one another. It is therefore likely that other family members like CRMP1 and CRMP5 might have compensated for the effects of CRMP2 in these experiments [37]. In dissociated cerebellar cultures, Purkinje cells are more fragile and have less cellular contacts compared to organotypic slice cultures or the in vivo situation, thus effects from inadequate CRMP2 phosphorylation will be more penetrant. Furthermore, CRMP2 itself has additional phosphorylation sites like the GSK3β target site Thr514 or the Cdk5 phosphorylation site Ser522, which are also important for dendritic development [28, 38, 39] and which might be partially overlapping in activity with the Thr555 site. We found that pSer522 levels were unchanged in PKCγ(S361G)-mice and after PMA treatment of OTSCs (Supplementary Fig. 2 c) and that Thr514 was only weakly increased (Supplementary Fig. 2 d), which is likely to be a downstream effect of Thr555 phosphorylation [40].

Taken together, our findings show that CRMP2 is a major target of PKCγ phosphorylation and an important effector of the reduction of dendritic growth seen after increased PKCγ activity.

## Electronic Supplementary Material

Supplementary Fig. 1 miRNA-mediated knockdown of CRMP2. a) HEK293 cells were transfected with CRMP2 and either CRMP2-specific miRNA or an unspecific control miRNA. b) CRMP2 expression is reduced in dissociated Purkinje cells either transfected with CRMP2-miRNA. Arrows show the Purkinje cell soma. Scale bar =25μm. (PNG 484 kb)

High resolution image (TIF 87769 kb)

Supplementary Fig. 2 CRMP2 phosphorylation in PKCγ(S361G)-mice. Immunoblots prepared from OTSCs of Wt- or PKCγ(S361G)-mice or Wt-cultures treated with PMA showing the expression of total CRMP2 (a), pThr555-CRMP2 (b), pS522-CRMP2 (c) or pThr514-CRMP2 (d). (PNG 308 kb)

High resolution image (TIF 68461 kb)

Supplementary Fig. 3 Examples of transfected cells for the overexpression of CRMP2 or its phospho-mimetic and phospho-defective mutants. Additional images of dissociated cerebellar cultures showing Purkinje cells expressing GFP. Cells were transfected with GFP (a) or GFP-tagged wildtype CRMP2 (b), phospho-defective (T555A) (c) or phospho-mimetic (T555D) (d) CRMP2 under a Purkinje cell specific promotor; Scale bars = 25 μm. (PNG 1262 kb)

High resolution image (TIF 209744 kb)

Supplementary Fig. 4 Examples of transfected cells for the knockdown of CRMP2. Additional images of transfected dissociated cultures using knockdown constructs containing the L7-promotor, a His-GST reporter and either a control miRNA (a) or CRMP2 specific miRNA (b). Scale bars = 25 μm. (PNG 628 kb)

High resolution image (TIF 100778 kb)

Supplementary Fig. 5 Examples of transfected CRMP2^ki/ki^ cultures transfected with wildtype CRMP2 or the T555D-mutant. Additional images of dissociated cultures from from CRMP2^ki/ki^-mice transfected with GFP only (a), GFP-CRMP2 (b) or GFP-T555D (c). Scale bars = 25 μm. (PNG 1290 kb)

High resolution image (TIF 207400 kb)

Supplementary Table 1 Report of results LC-MS/MS. Samples were prepared from either PKCγ(S361G)- or PKCγ^-/-^-mice. Antibodies against vGlut1, PKCγ or CRMP2 were used for the immunoprecipitation of the bait proteins. Total spectra of two sample duplicates are shown for the identified proteins. (XLSX 35.1 kb)

## Data Availability

All data and materials are presented within the article.
