# Supplementary figures and images for: PKCγ-Mediated Phosphorylation of CRMP2 Regulates Dendritic Outgrowth in Cerebellar Purkinje Cells
Source: Mol Neurobiol. 2020 Aug 29;57(12):5150–66. doi: 10.1007/s12035-020-02038-6 (PMC7541385; doi:10.1007/s12035-020-02038-6)

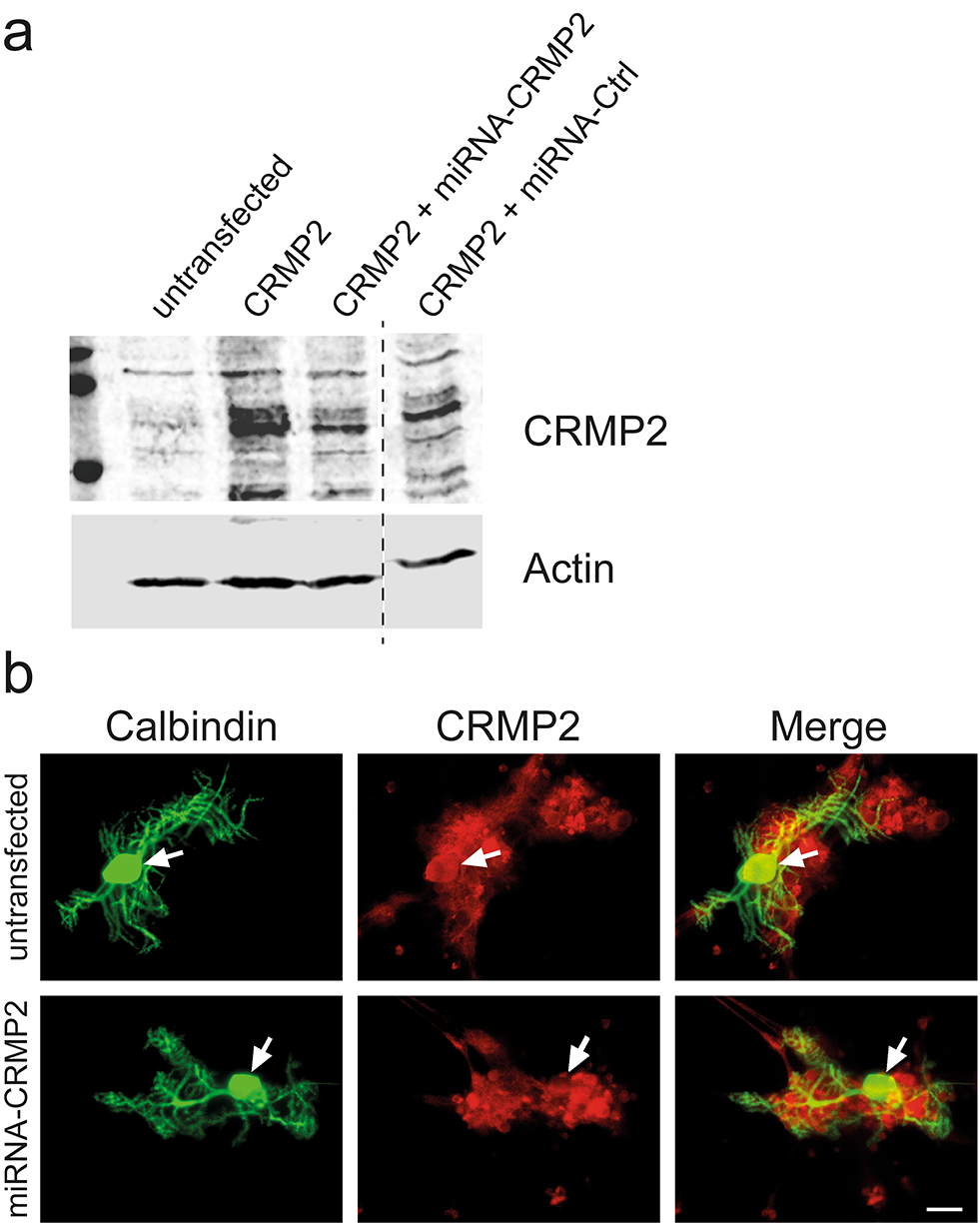

Supplement: Supplementary file 1 — miRNA-mediated knockdown of CRMP2. a) HEK293 cells were transfected with CRMP2 and either CRMP2-specific miRNA or an unspecific control miRNA. b) CRMP2 expression is reduced in dissociated Purkinje cells either transfected with CRMP2-miRNA. Arrows show the Purkinje cell soma. Scale bar =25μm. (PNG 484 kb) [file 12035_2020_2038_Fig9_ESM.png]

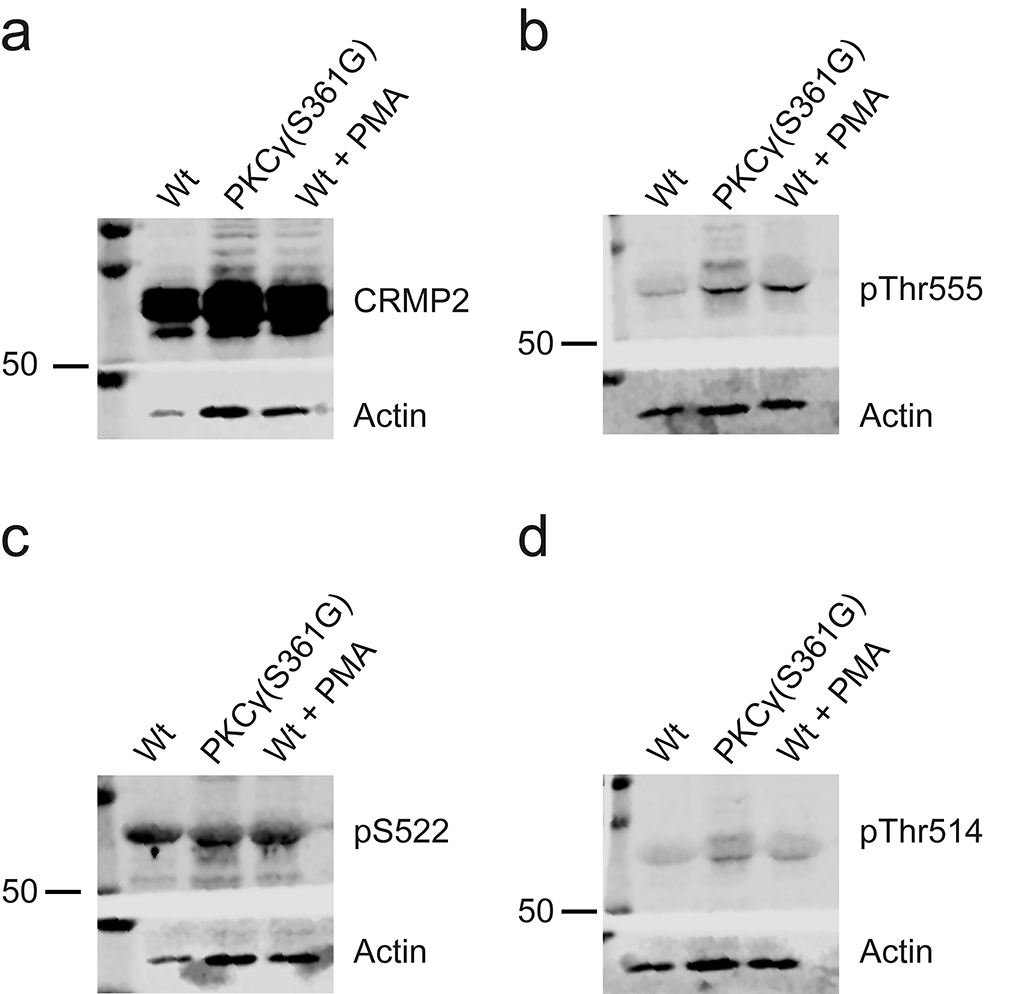

Supplement: Supplementary file 3 — CRMP2 phosphorylation in PKCγ(S361G)-mice. Immunoblots prepared from OTSCs of Wt- or PKCγ(S361G)-mice or Wt-cultures treated with PMA showing the expression of total CRMP2 (a), pThr555-CRMP2 (b), pS522-CRMP2 (c) or pThr514-CRMP2 (d). (PNG 308 kb) [file 12035_2020_2038_Fig10_ESM.png]

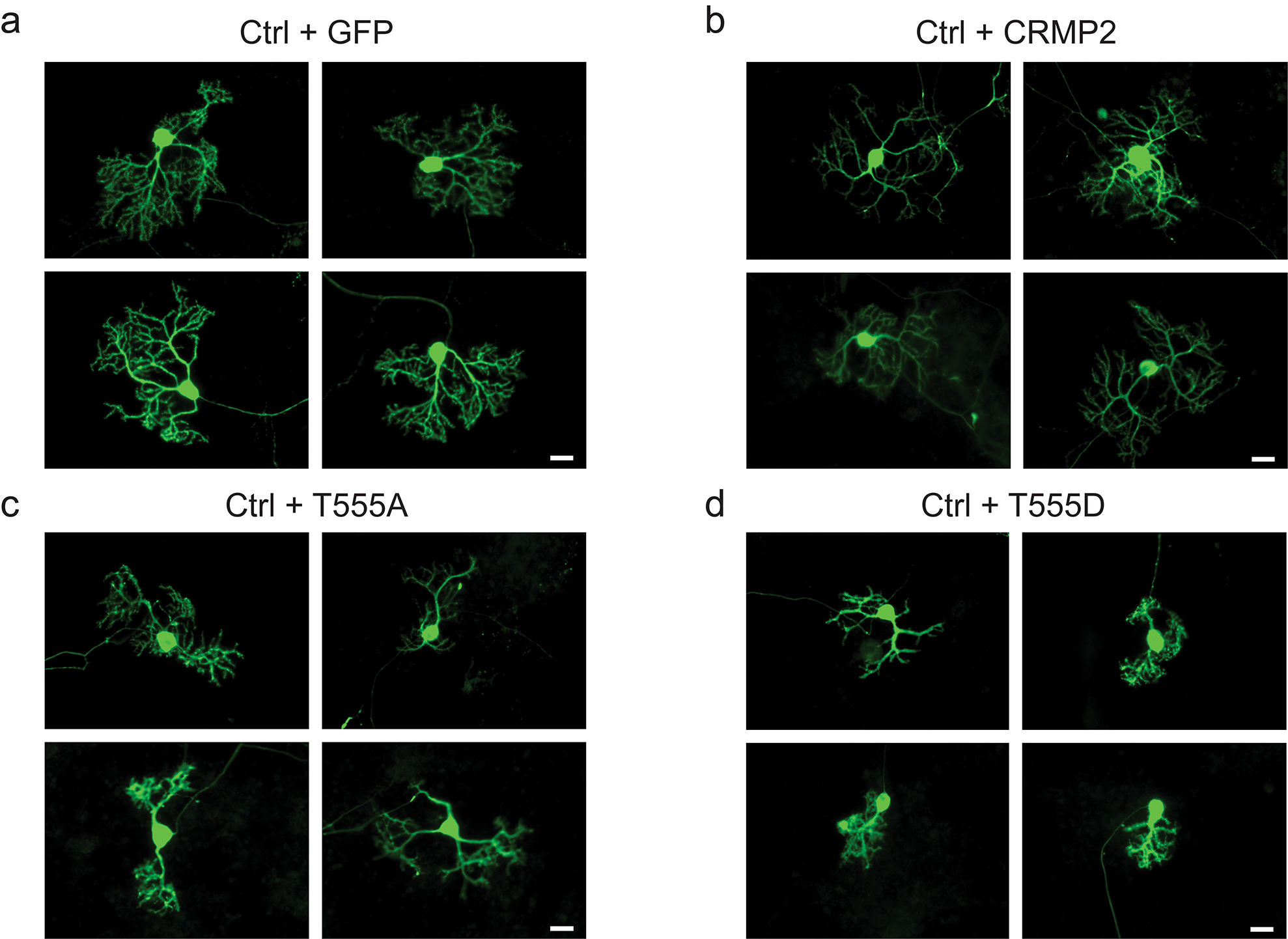

Supplement: Supplementary file 5 — Examples of transfected cells for the overexpression of CRMP2 or its phospho-mimetic and phospho-defective mutants. Additional images of dissociated cerebellar cultures showing Purkinje cells expressing GFP. Cells were transfected with GFP (a) or GFP-tagged wildtype CRMP2 (b), phospho-defective (T555A) (c) or phospho-mimetic (T555D) (d) CRMP2 under a Purkinje cell specific promotor; Scale bars = 25 μm. (PNG 1262 kb) [file 12035_2020_2038_Fig11_ESM.png]

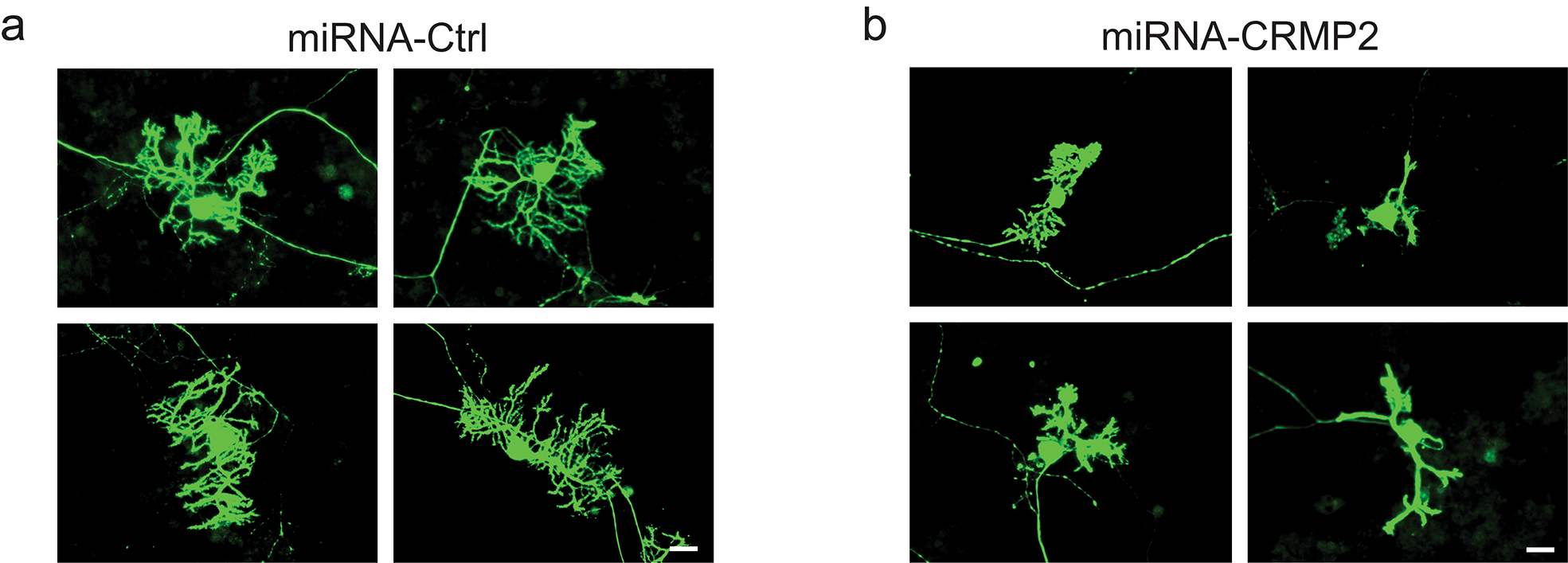

Supplement: Supplementary file 7 — Examples of transfected cells for the knockdown of CRMP2. Additional images of transfected dissociated cultures using knockdown constructs containing the L7-promotor, a His-GST reporter and either a control miRNA (a) or CRMP2 specific miRNA (b). Scale bars = 25 μm. (PNG 628 kb) [file 12035_2020_2038_Fig12_ESM.png]

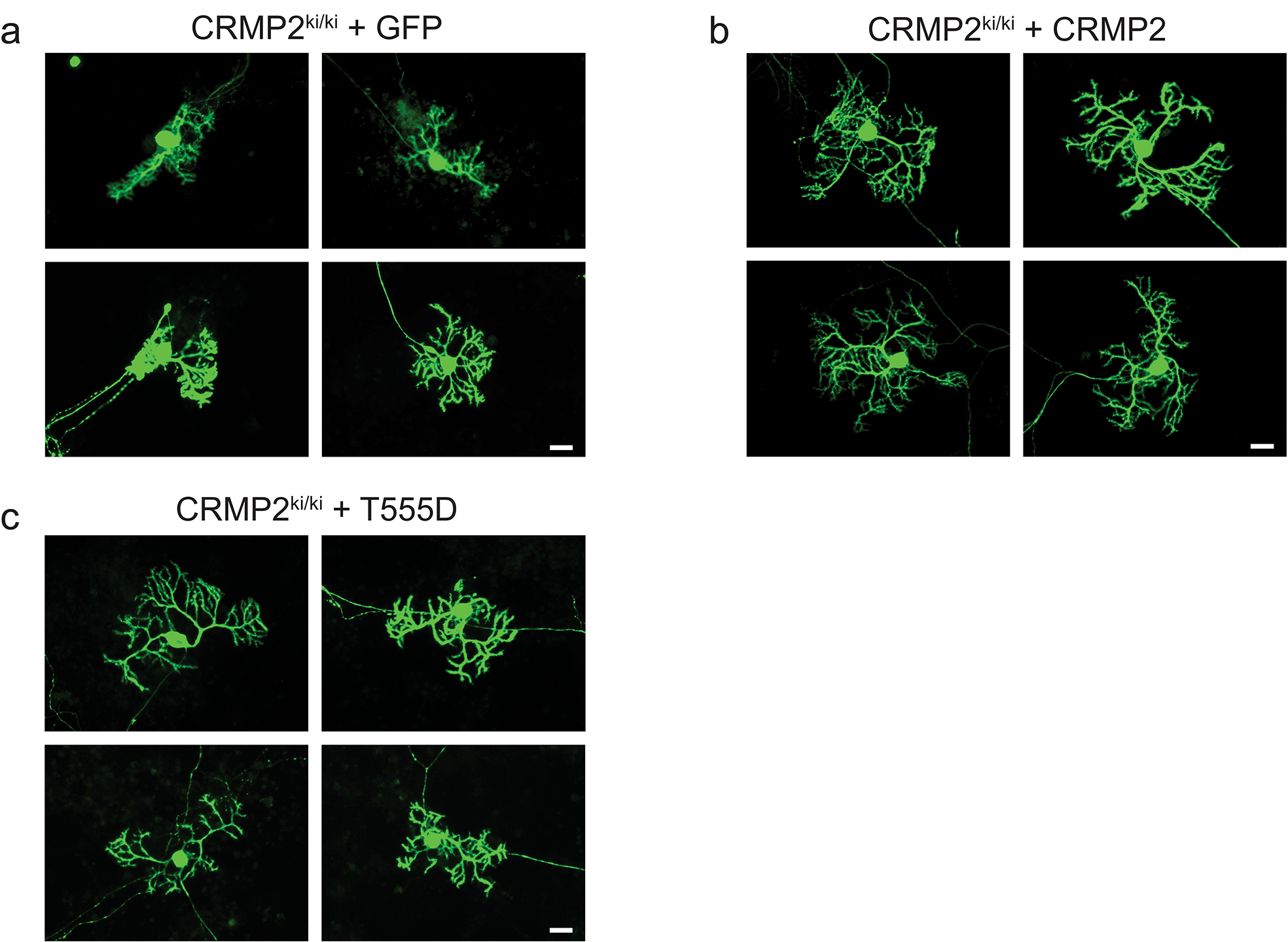

Supplement: Supplementary file 9 — Examples of transfected CRMP2ki/ki cultures transfected with wildtype CRMP2 or the T555D-mutant. Additional images of dissociated cultures from from CRMP2ki/ki-mice transfected with GFP only (a), GFP-CRMP2 (b) or GFP-T555D (c). Scale bars = 25 μm. (PNG 1290 kb) [file 12035_2020_2038_Fig13_ESM.png]
